# Supplementary material for: The genome sequence of the Uncertain moth, Hoplodrina octogenaria (Goeze, 1781)
Source: Wellcome Open Res. 2025 Apr 22;10:201. [Version 1] doi: 10.12688/wellcomeopenres.23983.1 (PMC12203046; doi:10.12688/wellcomeopenres.23983.1)
Supplement: Peer review history

# Reviewer report: Reviewer response for version 1

**DOI:** 10.21956/wellcomeopenres.26459.r123966

**Published:** 2025-06-26

**Authors:** Elizabeth Cash

**Affiliations:** 1 University of California Berkeley, Berkeley, California, USA; 2 University of Florida (Ringgold ID: 3463), Gainesville, Florida, USA

This manuscript presents a high-quality, chromosome-level genome assembly of the Uncertain moth, *Hoplodrina octogenaria*, using standard protocols from the Darwin Tree of Life initiative. The technical aspects of the work are well executed, and the assembly metrics, e.g., scaffold N50, chromosomal assignment, and BUSCO completeness, suggest a robust genomic resource. The methods are clearly described and replicable, and the dataset is accessible and valuable to the insect genomics community.

 To further enhance the impact of this work, the Background section would benefit from expanded framing to clarify the motivation for sequencing this species. Many genome announcements, including those associated with the Darwin Tree of Life initiative on Wellcome Open Research, include brief contextual information about the focal species’ biology and relevance. In this case, a concise overview of *H. octogenaria*’s ecological role, potential resilience in changing environments, or value in comparative genomic studies would help establish a clearer rationale for its inclusion in the project. In addition, the manuscript would be strengthened by the inclusion of a final Discussion section that helps synthesize the assembly results and place them in context with other Lepidoptera or noctuid genomes. This could include discussion of genome size, heterozygosity, repeat content, or other metrics, and highlight how this resource could support future research. Without this type of synthesis, the report reads more like a procedural summary than a cohesive genome announcement.

 The figures and tables are generally clear and informative, but a minor inconsistency should be addressed: the BUSCO “missing” (M) value differs between Table 2 (0.9%) and Figure 2 (1.1%), and one should be corrected to reflect the accurate value. Additionally, the manuscript notes that PacBio and Hi-C sequencing libraries were prepared from different individuals and from different tissue types (abdomen for long reads; head/thorax for Hi-C), but no rationale is provided for this choice. While this approach may be acceptable, particularly for small-bodied taxa, a brief explanation would improve transparency and help guide future efforts using similar methodologies. Given that PacBio sequencing was conducted on abdominal tissue, some of these sequences may represent biologically relevant symbionts or gut microbiota. Even if it’s not within the scope of the current study, retaining or documenting these data could be valuable for future comparative microbiome work or insect symbiosis studies. As such, if any bacterial or cobiont contigs were identified and excluded during the ASCC screening process, the authors may also consider summarizing and archiving these separately.

 Finally, although the manuscript is technically clear and follows the standard format used by the Darwin Tree of Life initiative, the writing style is somewhat fragmented. Many sections are composed of short or single-sentence paragraphs, with limited narrative flow or transitions. Consolidating related information into more cohesive paragraphs and adding a Discussion section would improve readability and enhance the manuscript’s overall coherence and scientific value.

Are sufficient details of methods and materials provided to allow replication by others?

Yes

Is the rationale for creating the dataset(s) clearly described?

Partly

Are the datasets clearly presented in a useable and accessible format?

Yes

Are the protocols appropriate and is the work technically sound?

Yes

Reviewer Expertise:

Genomics; functional genetics; entomology

I confirm that I have read this submission and believe that I have an appropriate level of expertise to confirm that it is of an acceptable scientific standard, however I have significant reservations, as outlined above.

# Reviewer report: Reviewer response for version 1

**DOI:** 10.21956/wellcomeopenres.26459.r124533

**Published:** 2025-06-13

**Authors:** Noah H Rose

**Affiliations:** 1 University of California San Diego, San Diego, California, USA

This report describes a genome assembly for the Uncertain moth. This methods used are appropriate and are described in sufficient detail to allow replication. This work will provide a valuable resource to the insect genomics community. The manuscript is clearly written and the work is done to a high standard. Assembly metrics indicate sufficient assembly quality to enable downstream research. Planned future annotation through the Ensembl pipeline will further enhance the utility of this resource.

Are sufficient details of methods and materials provided to allow replication by others?

Yes

Is the rationale for creating the dataset(s) clearly described?

Yes

Are the datasets clearly presented in a useable and accessible format?

Yes

Are the protocols appropriate and is the work technically sound?

Yes

Reviewer Expertise:

Genomics, evolutionary genetics, vector biology

I confirm that I have read this submission and believe that I have an appropriate level of expertise to confirm that it is of an acceptable scientific standard.

# Reviewer report: Reviewer response for version 1

**DOI:** 10.21956/wellcomeopenres.26459.r124527

**Published:** 2025-06-03

**Authors:** Shiping Liu

**Affiliations:** 1 Southwest University, Chongqing, Chongqing, China

This study conducted whole-genome sequencing of the male lepidopteran insect *Hoplodrina octogenaria* (family Noctuidae) and successfully assembled a 476.65 Mb nuclear genome (with 99.98% of sequences anchored into 31 chromosomal pseudomolecules. This high-quality chromosome-level genome provides essential foundational data for future research on species evolution and functional genomics.

 1. It is recommended to expand the Background section by incorporating key biological traits, ecological significance, and current research progress on *Hoplodrina octogenaria*. This will provide a stronger rationale for conducting genome sequencing, highlighting its necessity in advancing our understanding of this species' evolutionary adaptations, ecological interactions, and conservation needs. The abstract should also provide an overview of the research background.

 2. To improve readability, this article may benefit from a clearer structure, including distinct sections like Introduction (or Background), Materials and Methods, Results, Discussion, and Conclusion.

Are sufficient details of methods and materials provided to allow replication by others?

Yes

Is the rationale for creating the dataset(s) clearly described?

Partly

Are the datasets clearly presented in a useable and accessible format?

Yes

Are the protocols appropriate and is the work technically sound?

Yes

Reviewer Expertise:

Gene expression and regulation; functional genomics

I confirm that I have read this submission and believe that I have an appropriate level of expertise to confirm that it is of an acceptable scientific standard, however I have significant reservations, as outlined above.
